# Carcinome mucineux primitif cutané: à propos de deux cas et d'une revue de la literature

**DOI:** 10.11604/pamj.2014.18.340.2970

**Published:** 2014-08-27

**Authors:** Ihsane Souaf, Hassania Ameurtesse, Fatema Zehra Debbagh, Karima Idrissi, Kawter Znati, Affaf Amarti

**Affiliations:** 1 Service d'Anatomie et de Cytologie Pathologique, CHU Hassan II, Fès, Maroc; 2 Service de Dermatologie et Vénérologie, CHU Hassan II, Fès, Maroc; 3 Laboratoire de Recherche et Biologie de cancer, CHU Hassan II, Fès, Maroc

**Keywords:** Carcinome mucineux, tumeur annexielle, immunohistochimie, mucinous carcinoma, adnexal tumor, immunohistochemistry

## Abstract

Le carcinome mucineux primitif cutané est une tumeur annexielle rare, développée à partir des glandes sudoripares. Il se localise en générale à l'extrémité céphalique, surtout la région périorbitaire. Nous rapportons deux observations d'un carcinome mucineux primitif de la face et de la région axillaire, chez deux patients âgés de 60 ans. L’étude histologique montrait une prolifération tumorale dermohypodermique, faite de cellules organisées en cordons, en amas et en massif cribriformes, au sein d'une substance mucoïde. En immunohistochimie les cellules tumorales exprimaient la cytokératine 7, l'EMA et les récepteurs hormonaux. L'actine musculaire lisse a bien marqué les cellules myoépithéliales au niveau de la composante in situ dans un seul cas. Le carcinome mucineux primitif cutané est difficile à différencier d'une métastase mammaire ou digestive. La mise en évidence de carcinome in situ ou de cellules myoépithéliale est en faveur de l'origine cutanée primitive. C'est une tumeur à croissance lente, avec des métastases exceptionnelles. Le traitement est chirurgical et le taux de récidive est très élevé. Et à travers ces deux observations, les auteurs mettent en relief les principaux aspects cliniques, histologiques, thérapeutiques de cette entité avec une revue de la littérature.

## Introduction

Le carcinome mucineux primitif cutané est une tumeur annexielle rare des glandes sudoripares, décrite pour la première fois par Lennox et al. En 1952 [1], avec moins de 130 cas décrit dans la littérature à ce jour [2]. C'est une tumeur maligne de bas grade, avec un faible potentiel métastatique [3], et a croissance lente, mais dont l’évolution est émaillée par des récidives locales dans près de 50% des cas [4]. Des formes rapidement progressives ont été rapportées, mais le pronostic reste favorable. Il siège essentiellement au niveau de la tête et surtout la région périorbitaire de la paupière [5, 6], Il peut survenir à tout âge, mais prédomine après 60 ans [3]. Il est important d’éliminer une localisation secondaire avant de retenir une atteinte primitive [4]. Nous rapportons deux observations de carcinome mucineux primitif de la peau et discutons ses caractéristiques anatomo-clinique et immunohistochimique permettant de la distinguer des métastases cutanées d'un adénocarcinome mucineux.

## Patients et observations

**Figure 1:**
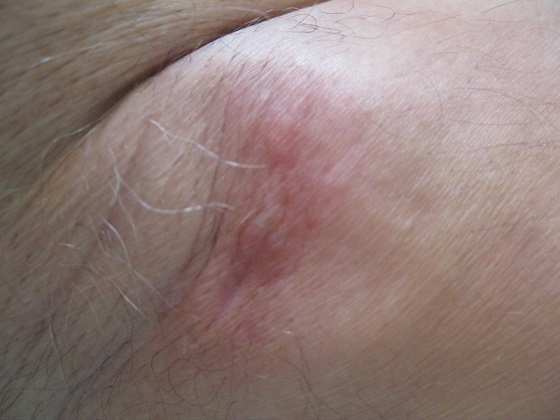
Tuméfaction axillaire, nodulaire, mal limitée, de couleur rouge. (observation 1)

**Figure 2:**
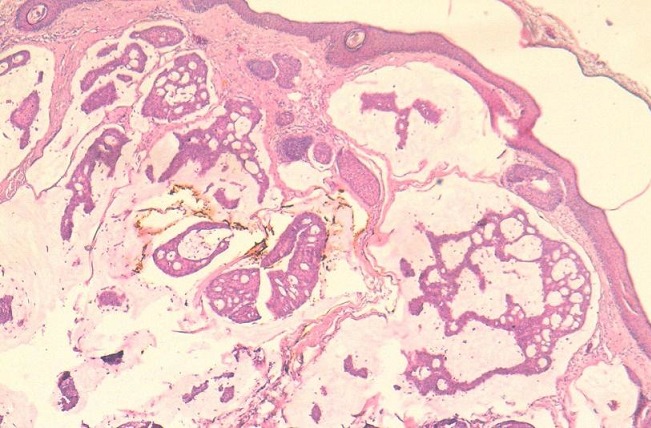
Architecture cribriforme, composée de cellules cohésives à noyau vésiculeux, à cytoplasme éosinophile parfois vacuolisé (HES x 20). (observation 1)

**Figure 3:**
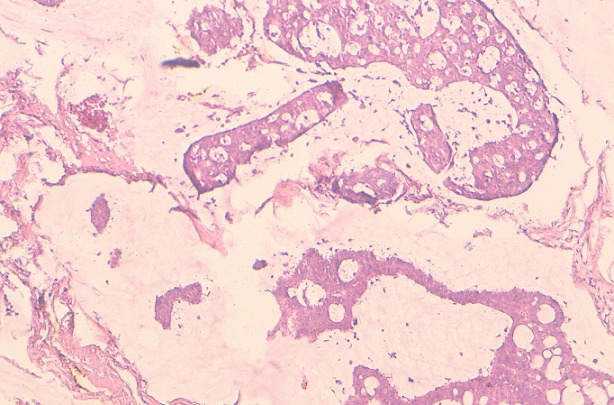
Prolifération dermique renfermant de larges plages de mucine, séparés par des cloisons fibreuses (HES x 5). (observation 1)

**Figure 4:**
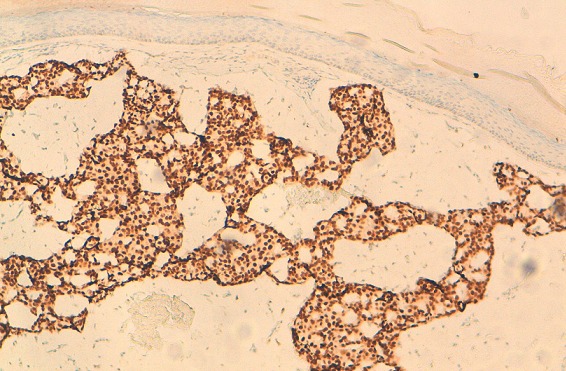
Immunomarquage positif de la Cytokératine 7 (HES x 20). (observation 1)

**Observation 1:** Il s'agit d'un patient âgé de 60 ans, sans ATCD pathologique notable, présentant une tuméfaction érythémateuse, axillaire gauche évoluant depuis un an et ½ (Figure 1). A l'examen clinique, cette tuméfaction était nodulaire, mal limitée, de couleur rouge, indolore, mesurant 2 cm. Une exérèse large de la tumeur a été réalisée, avec une recoupe profonde était réalisée. L’étude histologique a porté sur une prolifération tumorale carcinomateuse dermo-hypodermique, constituée de petites cellules régulières, rondes et cuboïdes, à noyaux basophiles vésiculeux, à cytoplasme éosinophile abondant, parfois vacuolisé (Figure 2). Elles se regroupent en formation glandulaire, trabéculaires et en massifs cribriformes, noyées dans des plages de stroma très clair, riche en mucine (Figure 3), colorée par le PAS (Periodic Acid Schiff) et séparées par des cloisons fibreuses. Les atypies cytonucléaires étaient discrètes et les mitoses rares. Les marges chirurgicales passaient en zone saine. L’étude en immunohistochimie montrait que les cellules tumorales exprimaient la cytokératine7 (Figure 4), l'EMA (Epithelial Membrane Antigen) et les récepteurs hormonaux. La cytokératine 20 était négative excluant une métastase d'origine digestive.

**Observation 2:** Nous rapportons le cas est un patient âgé de 60 ans, diabétique, avait consulté pour une tuméfaction de la joue gauche, évoluant depuis 4 ans et ayant augmentée progressivement de taille. A l'examen clinique, cette tuméfaction était nodulaire, bien limitée, de couleur chair, indolore, sans ulcération en regard, fixe par rapport au plan profond et mesurant 2,5 cm de diamètre. Une exérèse large de la tumeur a été réalisée, avec une recoupe profonde était réalisée.

**Figure 5:**
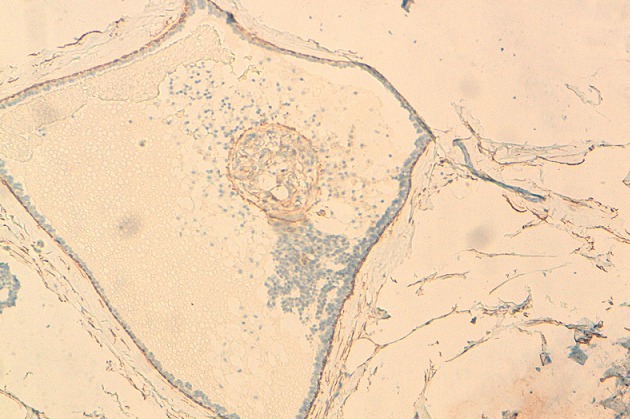
Immunomarquage positif de l'AML par les cellules myoépitheliales (HES x 40). (observation 2)

L'examen histologique et immunohistochimique était similaire à la première observation. Cette lésion comportait également une composante in situ marquait par l'actine musculaire (Figure 5). L'examen des seins et des aires ganglionnaires était sans particularités chez les deux patients. Le bilan d'extension chez les deux patients comprenait une radiographie thoracique, une échographie abdominale, une fibroscopie ‘sogastroduodénale et une colonoscopie révélait pas d'autre localisation. Suite à ces données histologiques, immunohistochimiques, radiologiques et fibroscopiques, le diagnostic de carcinome mucineux primitif cutané était retenu

## Discussion

Le carcinome mucineux primitif cutané est une tumeur annexielle très rare des glandes sudoripares. Il survient en moyenne entre 50 et 60 ans, avec des extrêmes allants de 8 à 84ans, avec une prédominance masculine (sex-ration de 2/1) [3]. Il touche la région cervico-faciale dans 80% des cas, notamment la face et le cuir chevelu avec une prédilection pour la région périorbitaire qui est atteinte dans 40% des cas, mais toute les localisations ont été rapportées [7]. L'origine apocrine ou eccrine est encore débattue. Pour certains auteurs, la différenciation dépendrait du siège, les carcinomes mucineux primitifs cutanés axillaires seraient de nature apocrine et ceux de la face seraient de nature eccrine [4].

Le carcinome mucineux primitif cutané présente une similitude morphologique avec les métastases cutanées d'un carcinome mucineux d'origine essentiellement mammaire ou gastro-intestinale [8]. Différencier un carcinome mucineux primitif cutané d'une métastase est histologiquement difficile. Ceci doit reposer sur une enquête clinique exhaustive, néanmoins quelques particularités histologiques et immunohistochimique permettent d'orienter vers la nature primitive [8]. Les métastases des adénocarcinomes colorectaux mucineux se localisent à la paroi abdominale, alors que les carcinomes mammaires atteignent la paroi thoracique [4]. Dans une vaste étude de métastases cutanées, Brownsten et al constaté que seulement 6% des cas avaient une métastase localisée au visage [4]. Certains auteurs se basent sur le fait que dans la tumeur secondaire, les cellules tumorales plus nombreuses et plus atypiques, les flaques de mucus sont peu représentées et les septas sont rares [8]. Sa présentation clinique habituelle est celle d'une lésion solitaire, asymétrique, nodulaire, de couleur chaire, indolore, d'une taille moyenne de 2cm et de croissance lente [8], mais des formes rapidement progressive ont été rapportés [4].

Histologiquement, c'est une tumeur dermohypodermique déparée par des cloisons fibreuses comportant des travées, des amas et des massifs cribriformes au sein d'une substance mucoïde. Les cellules tumorales sont cohésives, cubiques avec un cytoplasme parfois vacuolisé. Le noyau est central, vésiculeux et peu atypiques. Les mitoses sont rares ou absentes [3]. A l’étude immunohistochimique, le carcinome mucineux primitif cutané exprime les cytokératines de faible poids moléculaire, l'EMA, l'ACE (l'antigène carcinoembryonnaire), le GCDFP-15 (gross cystic disease fluid protein) et rarement la protéine S100, alors que la cytokératine 20 est toujours négative éliminant ainsi une origine digestive [3]. Certains auteurs ont rapportés une positivité des cellules carcinomateuses primitives pour les récepteurs hormonaux soulignant leurs similitudes avec les cellules carcinomateuses d'origine mammaire [8].

Nos cas exprimaient la cytokératine 7, l'EMA et la cytokératine 20 était négative. Qureshi et al suggèrent que la recherche d'une composante in situ oriente vers l'origine primitive annexielle cutanée, telle que des lésions d'hyperplasie intracanalaire atypiques au carcinome canalaire in situ [9]. La mise en évidence des cellules myoépithéliales par immunomarquage par la P63, la cytokératine 5/6 ou l'actine musculaire lisse peut être contributive au diagnostic [10]. La composante in situ a été observée en un seul cas et l'origine primitive a été retenue. En l'absence de cette composante in situ, la confrontation avec les données cliniques et paracliniques s'avère nécessaire [8].

Le traitement est chirurgicale, avec des marges larges, voir une chirurgie selon la technique de Mohs [11]. Le curage ganglionnaire régional est nécessaire s'il existe des adénopathies palpables. Nos patients ont bénéficié d'une exérèse large et les limites chirurgicales périphériques étaient saines. Le taux de récidive locale est de près de 50% [12] et les métastases à distance surviennent dans 2,7% [4]. Notre patient présente une forme classique de carcinome mucineux primitif cutané, traité avec succès par une exérèse élargie.

## Conclusion

Le carcinome mucineux primitif cutané est une tumeur maligne de bas grade à faible potentiel métastatique mais le taux de récidive est très élevé. Il est important de connaître cette entité car elle peut simuler une métastase d'origine mammaire ou digestive. La mise en évidence des cellules myoépithéliales ou d'une composante in situ est en faveur de la nature primitive, sinon la confrontation avec les données cliniques et paracliniques sera nécessaire.
